# Long non-coding RNA CCAT1 as a diagnostic and prognostic molecular marker in various cancers: a meta-analysis

**DOI:** 10.18632/oncotarget.24923

**Published:** 2018-05-04

**Authors:** Zhihui Zhang, Haibiao Xie, Daqiang Liang, Lanbing Huang, Feiguo Liang, Qiang Qi, Xinjian Yang

**Affiliations:** ^1^ Department of Spine Surgery, Shenzhen Second People's Hospital, The First Affiliated Hospital of Shenzhen University, Shenzhen 518039, China; ^2^ Shantou University Medical College, Shantou 515041, China; ^3^ Key Laboratory of Medical Reprogramming Technology, Shenzhen Second People's Hospital, The First Affiliated Hospital of Shenzhen University, Shenzhen 518039, China; ^4^ Department of Orthopaedics, Peking University Third Hospital, Beijing 100083, China

**Keywords:** long non-coding RNA, CCAT1, overall survival, lymph node metastasis, tumor node metastasis grade

## Abstract

**Purpose:**

Long non-coding RNA colon cancer-associated transcript-1 (CCAT1) is newly found to be related with diagnoses and prognosis of cancer. This meta-analysis was performed to investigate the relationship between CCAT1 expression and clinical parameters, including survival condition, lymph node metastasis and tumor node metastasis grade.

**Materials and Methods:**

The primary literatures were collected through initial search criteria from electronic databases, including PubMed, OVID Evidence-based medicine Reviews and others (up to May 12, 2017). Eligible studies were identified and selected by the inclusion and exclusion criteria. Data was extracted and computed into Hazard ratio (HR) for the assessment of overall survival, subgroup analyses were prespecified based on the digestive tract cancer or others. Analysis of different CCAT1 expression related with lymph node metastasis or tumor node metastasis grade was conducted. Risk of bias was assessed by the Newcastle-Ottawa Scale.

**Results:**

9 studies were included. This meta-analysis showed that high CCAT1 expression level was related to poor overall survival, the pooled HR was 2.42 (95% confidence interval, CI: 1.86-3.16; *P* < 0.001; fix- effects model), similarly in the cancer type subgroups: digestive tract cancer (HR, 2.42; 95% CI, 1.79–3.29; *P* < 0.001; fix- effects model) and others (HR, 2.42; 95% CI, 1.42–4.13; *P* = 0.001; fix- effects model). The analysis showed that high CCAT1 was strongly related to positive lymph node metastasis (Odds ratio, OR: 3.24; 95% CI, 2.04-5.16; *P* < 0.001; fix- effects model), high tumor node metastasis stage (OR, 3.87; 95% CI, 2.53–5.92; *P* < 0.001; fix- effects model).

**Conclusions:**

In conclusion, this meta-analysis revealed that CCAT1 had potential as a diagnostic and prognostic biomarker in various cancers.

## INTRODUCTION

Cancers become a major public health problem with a leading cause of morbidity and mortality [1]. Although with the new and systemic therapies used for cancer, patients suffer from an enormous financial burden and the benefit is relatively small [2]. Delays in the diagnosis of cancer, patients unfortunately miss optimal opportunity for treatment, have poorer quality of life, less chance of surviving and more expensive costs [2–4]. Therefore, to explore a way for early detection of cancer is critical to prognosis and treatment.

Recent advances in epigenetic research have found that epigenetic regulation activates the development of cancer [5]. Detection of early epigenetic changes is an attractive tool to screen and diagnose cancer early, with early appearance of epigenetic alternations and their potential biomarkers in cancer [6]. Studies have showed that epigenetic alternations in various cancer result from epigenetic activated processes of long non-coding RNAs (lncRNAs) [7]. LncRNAs are often described as non-coding transcripts more than 200 nucleotides in length [8]. LncRNAs expression profiles are recognized as signals of cellular states and programs especially in cancer [9]. In recent years, colon cancer-associated transcript-1 (CCAT1) is a rising star of oncogenic lncRNAs associated with various cancers.

CCAT1 activates cancer cell proliferation, migration and invasion, with 2628 base pairs in length located on chromosome 8q24.21 [10]. The expression of CCAT1 is significantly higher in cancer samples compared with adjacent normal samples [11]. The overexpression of CCAT1 has been identified as key activated in initiation and progression of cancers, such as cholangiocarcinoma (CCA) [12], esophageal squamous cell carcinoma (ESCC) [13], hepatocellular carcinoma (HCC) [14–16], melanoma (MEL) [17], gastric cancer (GC) [18], breast cancer (BRC) [19], colon cancer (CC) [20]. The deregulation of CCAT1 is closely related to clinical parameters, including overall survival (OS), lymph node metastasis (LNM) and tumor node metastasis (TNM) grade. CCAT1 have been found to be consistently overexpressed in relevant cancers and may serve as a prognostic biomarker to evaluate clinical outcomes. Recently most studies about CCAT1 just have reported a unique type of cancer, however, there have not been any studies systemically to review the relationship between deregulation of CCAT1 and clinical prognosis. We therefore perform the meta-analysis to evaluate clinical prognosis for different CCAT1 expression in cancers.

## MATERIALS AND METHODS

### Literature search

The relevant studies were searched from electronic databases of PubMed, OVID Evidence-based medicine Reviews, Cochrane Library and China National Knowledge Infrastructure (CNKI) in May 2017. The following terms were used: “CCAT1 or colon cancer associated transcript-1” and “cancer or tumor or carcinomas or neoplasm”. The last update of searching time was May 12, 2017. All full-text articles were obtained according to citation lists of retrieved articles.

### Inclusion and exclusion criteria

#### Inclusion criteria

To be included in this meta-analysis, studies had to met the following criteria: (1) Studies expressing an association between different expression levels of CCAT1 and prognosis of cancer patients; (2) Cancer patients was confirmed pathologically and never undergone chemotherapy and radiotherapy; (3) Relevant clinical variables such as LNM, TNM and OS for outcomes were reported; (4) Relevant data was extractable to perform the meta-analysis.

### Exclusion criteria

Exclusion criteria are as the following: (1) Studies failed to meet the inclusion criteria; (2) Editorials, letters, case reports, expert opinions, abstracts and reviews, animal experiment studies; (3) Duplicate publications.

### Data extraction

From the included studies, data was collected and organized independently by two of authors (Zhihui Zhang, Haibiao Xie), divergences were come to an agreement by another two of authors (Qiang Qi, Xinjian Yang). The primary characteristics were collected from each included study: cancer types, total numbers of patients, number of patients in high CCAT1 expression group or in low CCAT1 expression group, number of patients with positive LNM or negative LNM, number of patients in high TNM stage (HTS) or in low TNM stage (LTS), survival analysis, hazard ratios (HRs) and corresponding 95% CI for OS. In the absence of HR, the data was extracted from relevant figures of survival curve (SC). The secondary characteristics as follows: first author, publication date, country of origin, detection method and cut-off of CCAT1 expression levels, multivariate analysis and follow-up months.

### Quality assessment

Quality of primary studies was assessed by the Newcastle-Ottawa Scale (NOS), a score of 0-8 was evaluated each studies. Six or more scores was of high quality. All of these were carried out independently by two of authors (Daqiang Liang and Feiguo Liang), any disagreement was resolved in conference by two of authors (Lanbing Huang and Xinjian Yang).

### Statistical methods

Chi-square test was used to evaluate the heterogeneity for HRs between studies. If I^2^ < 50 and P(H) > 0.1, there was in absence of heterogeneity between studies, thus, the fixed-effects model was adopted. In contrast, the random-effects model was applied. Pooled HR with 95% confidence interval (CI) was calculated to describe survival situation comparing high CCAT1 expression group and low CCAT1 expression group. Funnel plots and Egger's test were adopted to assess the potential publication bias. Sensitivity analysis was performed to test the stability of the meta-analysis results. Furthermore, analysis of different CCAT1 expression related with LNM or TNM stage was conducted. Meta-analysis is performed by using the Stata12.0 with the level of significance set at 0.1%.

## RESULTS

### Studies selected

**Figure 1:**
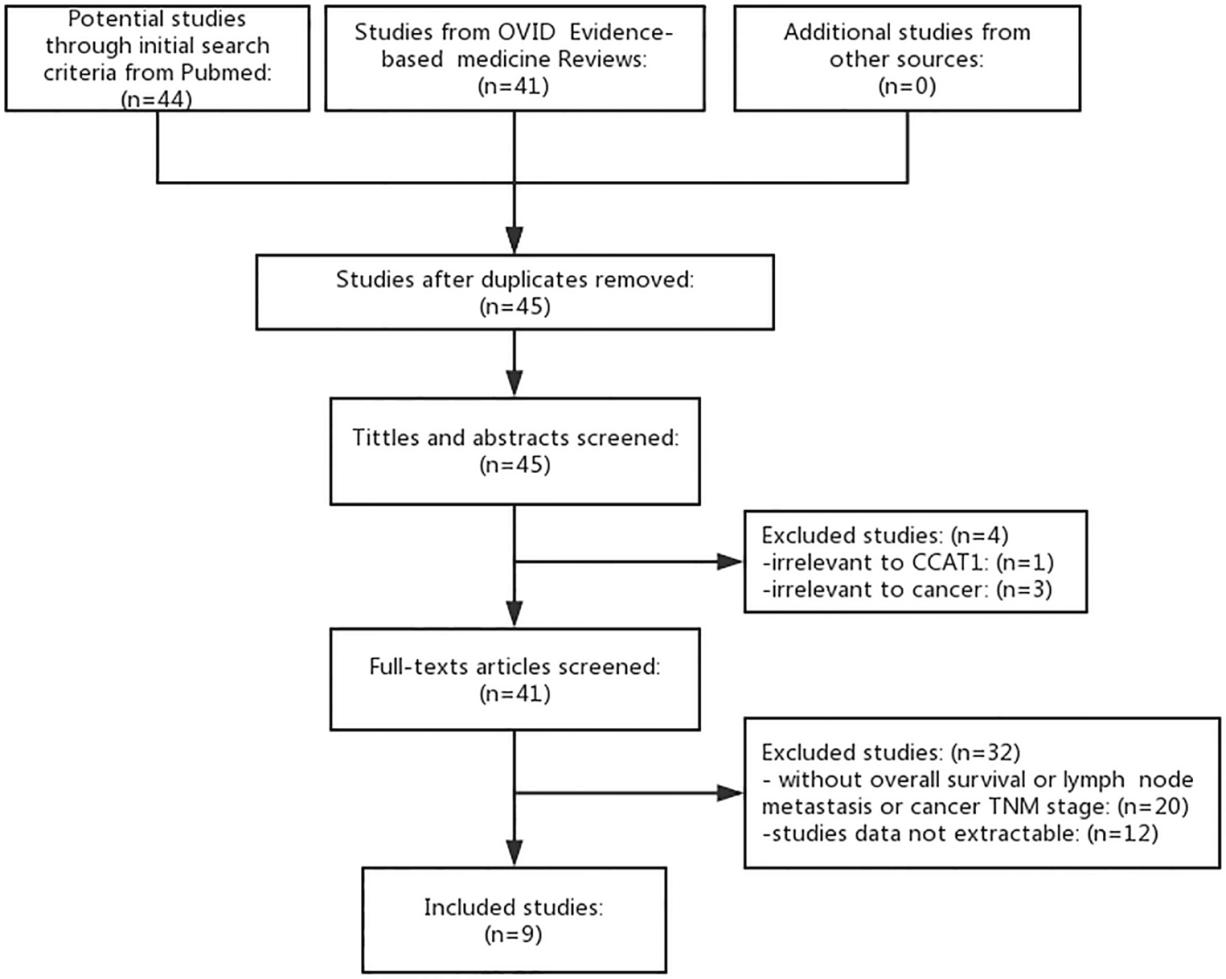
Flowchart of studies searched, selected, included and excluded The other sources means Cochrane Library and China National Knowledge Infrastructure (CNKI).

Forty-five articles were identified individually which met the initial search criteria. With all full-texts articles screened, we included nine eligible articles in the final analysis (Figure 1). The exclusions didn't cover extractable data of survival outcomes, LNM or TNM stage.

### Characteristics of included studies

These nine studies published time were from 2014 to 2017, which were all from China. A total of 573 patients were included (range from 30 to 92). Not all these studies covered all of OS, LNM and TNM. Among these nine studies, seven studies covered OS, five studies covered LNM, six studies covered TNM. There were seven different types of cancer in this meta-analysis, including digestive tract cancers (1CCA, 1ESCC, 3HCC, 1GC, 1CC) and other types of cancers (1melanoma, 1BRC), all of patients were diagnosed definitely based on pathology. Expression levels of CCAT1 in these patients’ cancer tissue were detected by use of qRT-PCR, thus, patients were divided into two groups: high and low expression of CCAT1. Both of groups contained two subgroups: LNM group and TNM group. As for the absence of reporting HR statistic, we acquired HRs from relevant figures of SC (Supplementary Table 1). Almost all the included studies had low risk of bias through assessment by using Newcastle-Ottawa Scale (Table 1).

**Table 1:** NOS for risk of bias assessment of included studies

Study	Selection				Comparability		Outcome		Total
	Adequacy of case definition	Number of case	Representativeness of the cases	Ascertainment of exposure	Ascertainment of detection method	Ascertainment of cut-off	Assessment of outcome	Adequate follow-up	
Jiang, X.M. [12]	1	1	1	1	1	1	1	1	8
Zhang, E. [13]	1	1	1	1	1	1	1	1	8
Dou, C.Q [14]	1	0	1	1	1	1	1	0	6
Lv, L. [17]	1	0	1	1	1	1	1	1	7
Zhou, B.G. [18]	1	0	1	1	1	1	1	0	6
Zhang, X.F. [19]	1	1	1	1	1	1	1	1	8
Zhu, H.Q [15]	1	1	1	1	1	1	1	1	8
Deng, L. [16]	1	1	1	1	1	1	1	1	8
He, X. [20]	1	0	1	1	1	1	1	1	7

NOS = Newcastle-Ottawa Scale.

### Meta-analysis result

#### The correlation between CCAT1 and OS in seven types of cancers

**Figure 2:**
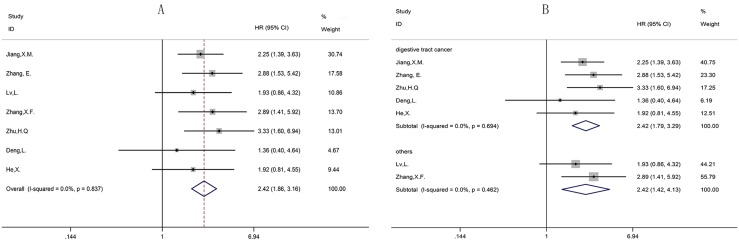
Forest plot of the correlation between HRs of OS and CCAT1 expression levels (**A**) Forest plot of the correlation between HRs and CCAT1 expression levels in different cancer patients. (**B**) Forest plot of the subgroup analysis for HRs by the factor of cancer types. HRs = Hazard ratios; OS = overall survival.

We obtained HRs for OS from seven studies incorporating 503 patients in seven types of cancers. There was no evidence of significant heterogeneity among the studies, thus, we use the fixed-effects model (Figure 2A). The Pooled HR was 2.42 (95% CI, 1.86–3.16; *P* < 0.001) that revealed a statistically significant negative correlation between expression levels of CCAT1 and OS. It was similar in the cancer type subgroups: digestive tract cancers (HR, 2.42; 95% CI, 1.79–3.29; *P* < 0.001) and others (HR, 2.42; 95% CI, 1.42–4.13; *P* = 0.001(Figure 2B)). It was statistically significant that OS had the benefit of low expression of CCAT1, in contrast, high expression of CCAT1 meant poor OS (Table 2).

**Table 2:** Results of this meta-analysis

Outcome	No.of studies	No.of patients	HR/OR (95% CI)	*P*	Heterogeneity	
					I^2^(%)	*p*-value
OS	7	503	2.422 (1.858–3.157)	< 0.001	0	0.837
DTC [12, 13, 15, 16, 20]	5	381	2.423 (1.786–3.288)	< 0.001	0	0.694
Others [17, 19]	2	122	2.418 (1.416–4.129)	= 0.001	0	0.462
LNM [12–14, 19, 20]	5	361	3.244 (2.039–5.160)	< 0.001	0	0.711
TNM [12–14, 18–20]	6	391	3.870 (2.529–5.923)	< 0.001	21.3	0.273

OS: overall survival; LNM: lymph node metastasis; TNM: cancer TNM stage; DTC: digestive tract cancer; Others: other cancer types; HR: hazard ratios; OR: odds ratios; No: number; CI: confidence interval.

### The correlation between CCAT1 and LNM

**Figure 3:**
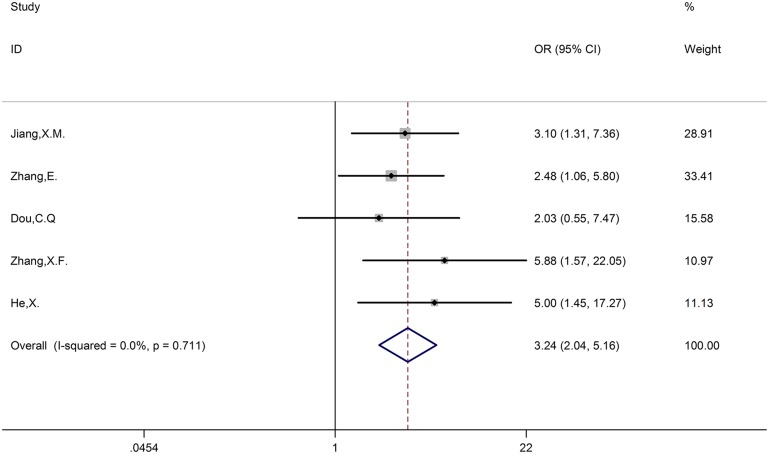
Forest plot of the correlation between LNM and CCAT1 expression levels LNM = lymph node metastasis.

Five studies incorporating 361 patients reported the association between patients with positive LNM or negative LNM and different expression levels of CCAT1. There was no evidence of significant heterogeneity among the studies, thus, we use the fixed-effects model. The odds ratio (high CCAT1 expression group versus low CCAT1 expression group) was 3.24 (95% CI, 2.04–5.16; *P* < 0.001). The outcome showed that patients with positive LNM were associated with high CCAT1 expression, whereas patients with negative LNM were associated with low CCAT1 expression (Figure 3).

### The correlation between CCAT1 and TNM

**Figure 4:**
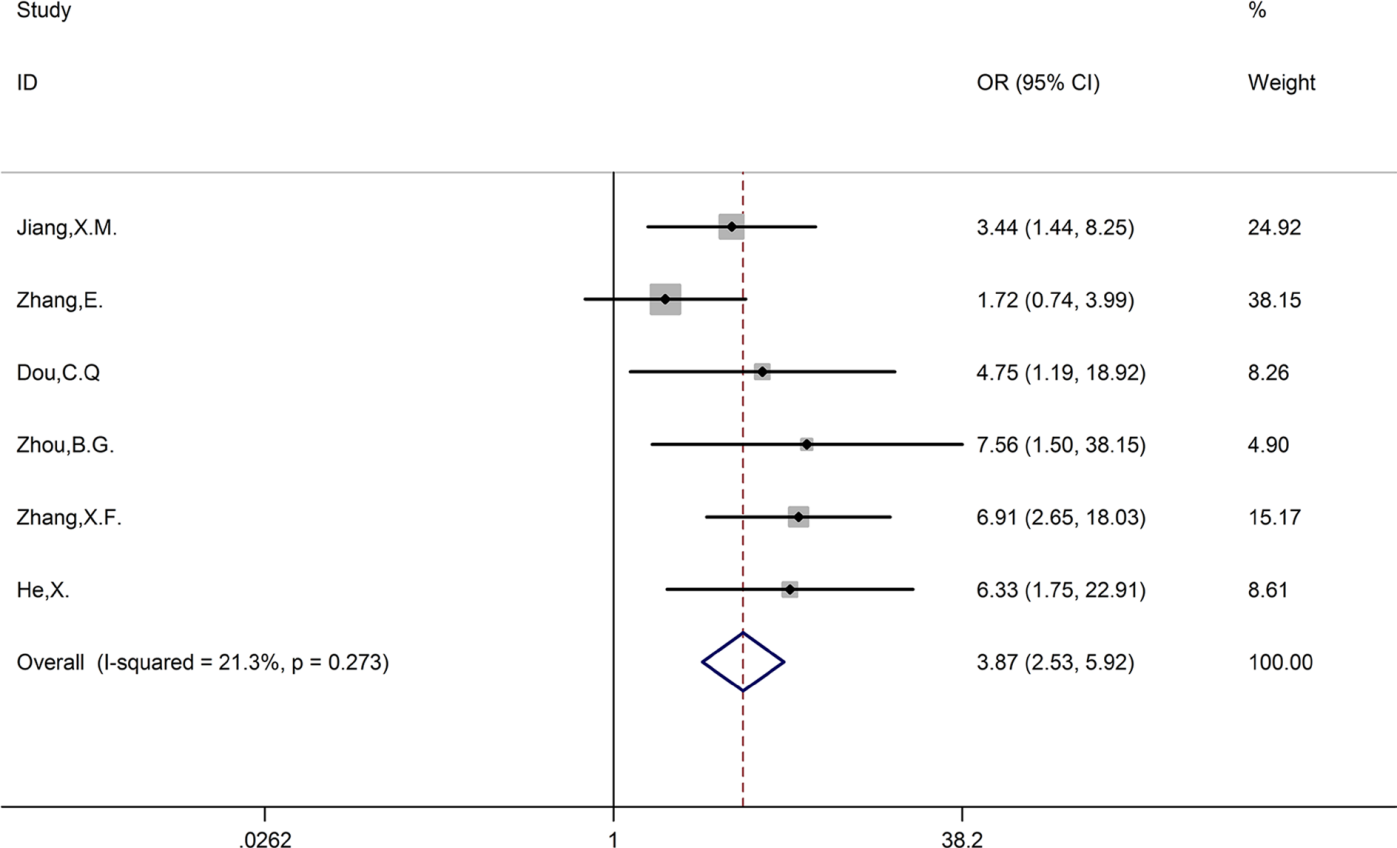
Forest plot of the correlation between TNM stage and CCAT1 expression levels

Six studies incorporating 391 patients reported the association between patients in different TNM stages and expression levels of CCAT1. There was no evidence of significant heterogeneity among the studies, thus, we use the fixed-effects model. The odds ratio (high CCAT1 expression group versus low CCAT1 expression group) was 3.87 (95% CI, 2.53–5.92; *P* < 0.001). The result showed that patients in high TNM stage (HTS) were associated with high CCAT1 expression, whereas patients in low TNM stage (LTS) were associated with low CCAT1 expression (Figure 4).

### Sensitivity analysis and publication bias

**Figure 5:**
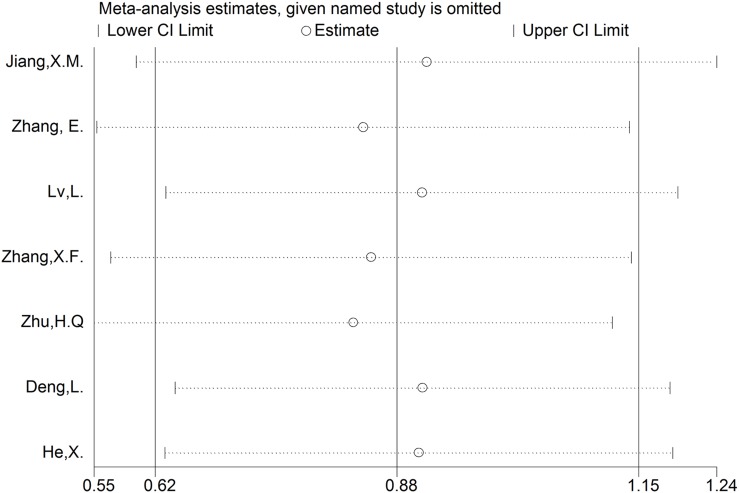
Sensitivity analysis of effect of each study on the pooled HRs

**Figure 6:**
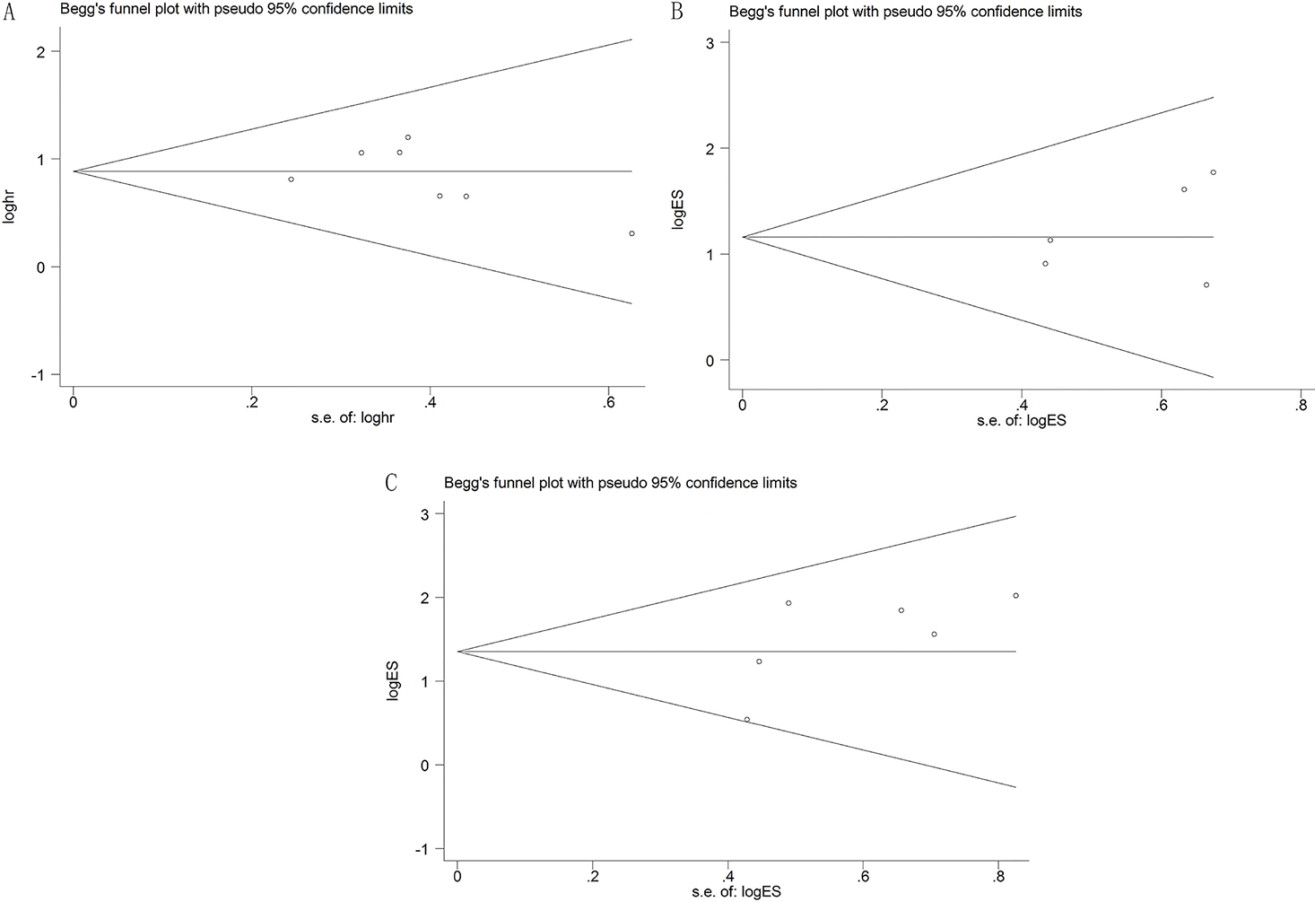
Funnel plots of potential publication bias (**A**) in OS group; (**B**) in LNM group; (**C**) in TNM group.

**Figure 7:**
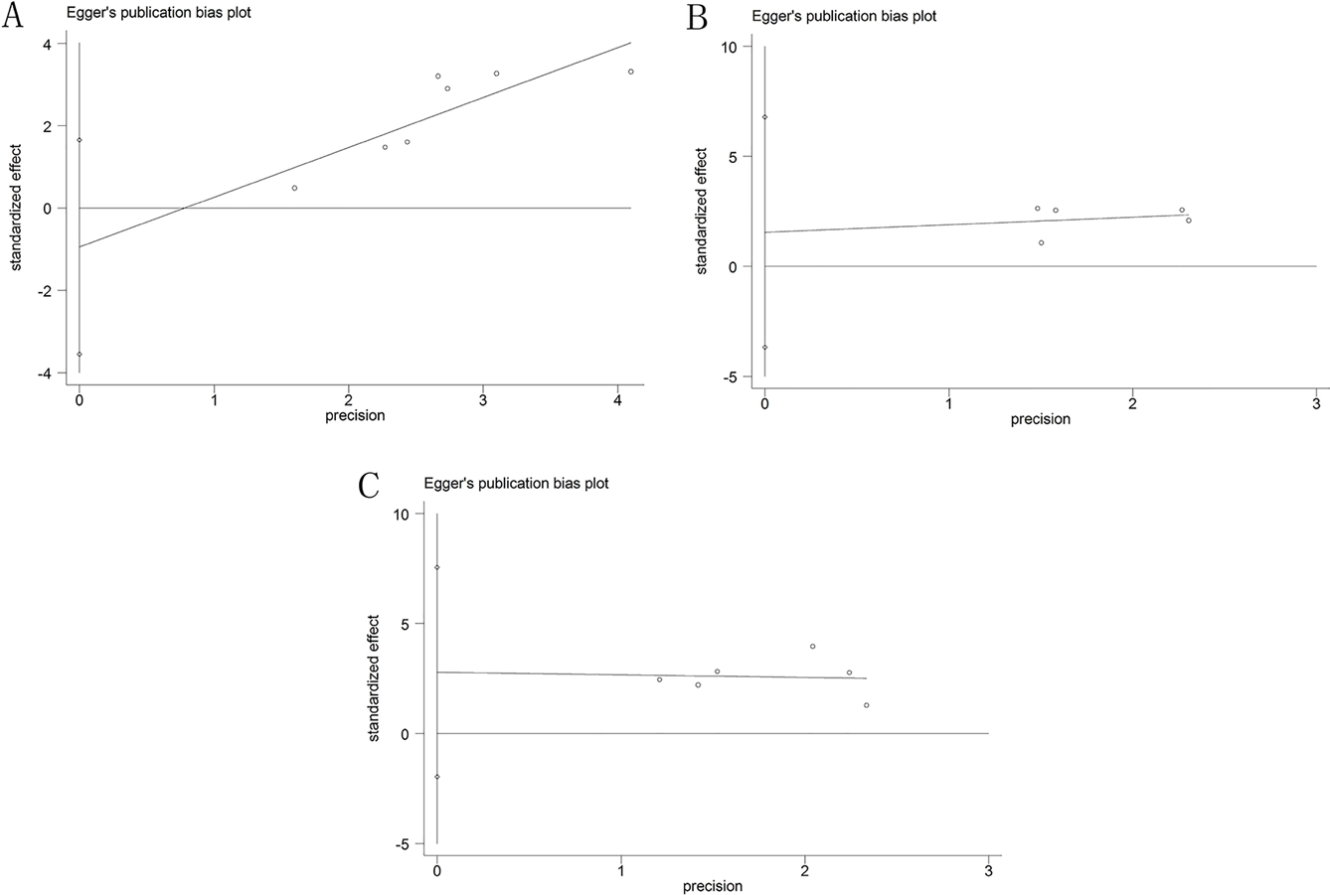
Egger's plot of potential publication bias (**A**) in OS group; (**B**) in LNM group; (**C**) in TNM group.

Sensitivity analysis was used to test the stability of the meta-analysis results of CCAT1 and OS. Consequently, the result pattern was not significantly impacted (Figure 5). The Funnel plots and Egger's test were conducted to evaluate the publication bias (Figures 6, 7). The result indicated no obvious publication bias in this meta-analysis: p(OS) = 0.395, p(LNM) = 0.417, p(TNM) = 0.179; t(OS) = -0.93, t(LNM) = 0.94, t(TNM) = 1.63.

## DISCUSSION

CCAT1, a newly discovered lncRNA, could activate cancer cell proliferation, migration and invasion and its overexpression is correlated with poor clinical outcomes [10]. We performed this meta-analysis included nine studies (involving seven types of cancer) to investigate clinical parameters of CCAT1. The pooled analysis indicated patients were at risk of poor survival, lymph node metastasis and high tumor stage, when the expression of CCAT1 was high. Therefore, our results showed that CCAT1 probably could serve as a diagnostic and prognostic biomarker in various cancers.

High expression levels of some lncRNAs have already been associated with poor prognosis in various types of cancer [21]. Likewise, CCAT1 had the same conclusion. As shown in Figure 2, we observed a statistically significant negative correlation between expression levels of CCAT1 and OS without obvious publication bias. Most of types of included cancers were digestive tract cancers, so we divided cancers into two groups digestive tract cancer and others to investigate the independent role in cancers. The result showed that CCAT1 was an independent prognostic factor for various cancers. As for LNM group and TNM group, we found that LNM or TNM was positively correlated with expression levels of CCAT1 in absence of obvious publication bias (Figures 3, 4). To conclude, CCAT1 might serve as an oncogenic lncRNA and could be a detectable diagnostic and independent prognostic biomarker for various cancers.

Furthermore, the relationship between CCAT1 expression and prognosis suggested its potential utilities as a clinical tool to detect cancers early. Likewise, a lot of previous meta-analyses investigated the relationship between the other lncRNAs expression and prognosis of corresponding cancers, such as HOTAIR [22], H19 [23] and MALAT1 [24]. These studies referred interrelated pathways that could influence processes of cancers. To explore deeper resource of CCAT1 on cancer, we further reviewed CCAT1 and its potential targets, pathways and related microRNAs, which had its potential utilities as a target therapy (Table 3).

**Table 3:** Summary of CCAT1 with their potential targets, pathways and related microRNAs

Potential targets	Pathways	Related microRNAs	Reference
Bcl-xl	proliferation	let-7c	[25]
BMI1	proliferation, migration and invasion	miR-218	[26–28]
c-Myc	proliferation, migration and invasion	let-7, miR-155	[16, 20, 29–35]
CPEB2	miR-181a/CPEB2 axis	miR-181a	[36]
HMGA2	proliferation and migration	let-7	[16, 29]
hnRNPA1	CCAT1/miR-490 axis	miR-490	[14, 18]
HOXB13 SPRY4	growth and migration	miR-7	[13]
NA	cell proliferation and invasion	miR-33a	[17]
NA	proliferation	miR-410	[37]
P53	proliferation, migration and invasion	NA	[35]

The strength of this meta-analysis is that we systemically reviewed the relationship between CCAT1 expression and clinical parameters, including OS, LNM and TNM. In addition, we designed two groups according to types of cancers: the digestive tract cancer and others.

However, there are also several limitations in the present meta-analysis as follows. Firstly, although we have exhausted to research relevant studies, at present only nine studies are eligible to have been included in this meta-analysis. Secondly, not all of included studies have OS, LNM and TNM and other studies didn't provided detailed information, resulting a less sample size of studies to analyze. Thirdly, four studies do not provide exact HR, it is undetected whether bias occurs or not when we obtain HR from the OS curve. Fourthly, all included studies are from China, so it is not known whether our results are applicable to other countries. Although the other countries have widely investigated and reported CCAT1 from the basic research side, there are few studies to express an association between different expression levels of CCAT1 and prognosis of cancer patients. Recently, more and more Chinese focus on lncRNAs and try to find a biomarker to dectect cancers early. Fifthly, the cut-off value of CCAT1 expression level in respective study has its own criteria (median or mean), the consistent measurement standards is not existing. Therefore, it is necessary to confirm our results using a large size of studies and better design studies.

In conclusion, this meta-analysis summarizes the accumulating evidence that suggests CCAT1 potential as a diagnostic and prognostic biomarker in various cancers. It is of great benefit to the clinical diagnosis, furthermore, it probably leads to new drugs for retarding the progress of cancers. Our results imply that high CCAT1 expression is related to poor OS, LNM and TNM, but the quantitative data on CCATA expression is needed to test.

## SUPPLEMENTARY MATERIALS TABLE


