# Supplementary material for: Long non-coding RNA CCAT1 as a diagnostic and prognostic molecular marker in various cancers: a meta-analysis
Source: Oncotarget. 2018 May 4;9(34):23695–703. doi: 10.18632/oncotarget.24923 (PMC5955114; doi:10.18632/oncotarget.24923)
Supplement: Supplementary file 1 [file oncotarget-09-23695-s001.pdf]

Long non-coding RNA CCAT1 as a diagnostic and prognostic molecular marker in various cancers: a meta-analysis

SUPPLEMENTARY MATERIALS

Supplementary Table 1: Characteristics of included studies

Study (First author)	Year	Country	Cancer type	Total number	Detection method	Cut-off	CCAT1 expression										Survival analysis	Multivariate analysis	HR statistic	Hazard ratios (95%CI)	Follow-up months
								LNM		TNM			LNM		TNM						
							Low	Low with LNM	Low without LNM	Low in LTS	Low in HTS	High	High with LNM	High without LNM	High in LTS	High in HTS					
Jiang XM [12]	2017	China	CCA	91	qRT-PCR	Median	44	19	25	25	19	47	33	14	13	34	OS	Yes	Rep	2.250 (1.395–3.630)	50 (Total)
Zhang E [13]	2017	China	ESCC	90	qRT-PCR	Mean	45	19	26	23	22	45	29	16	17	28	OS	Yes	SC	2.88 (1.53–5.43)	60 (Total)
Dou CQ [14]	2017	China	HCC	40	qRT-PCR	Median	15	7	8	9	6	25	16	9	6	19	NA	No	NA	NA	NA
Zhu HQ [15]	2015	China	HCC	86	qRT-PCR	Median	43	NA	NA	NA	NA	43	NA	NA	NA	NA	OS	Yes	Rep	3.33 (1.60–6.96)	40 (Total)
Deng L [16]	2015	China	HCC	66	qRT-PCR	Median	33	NA	NA	NA	NA	33	NA	NA	NA	NA	OS	No	SC	1.36 (0.40–4.65)	30 (Total)
Lv L [17]	2017	China	MEL	30	qRT-PCR	Median	15	NA	NA	NA	NA	15	NA	NA	NA	NA	OS	No	SC	1.93 (0.86–4.30)	60 (Total)
Zhou BG [18]	2016	China	GC	30	qRT-PCR	Median	15	NA	NA	11	4	15	NA	NA	4	11	NA	No	NA	NA	NA
Zhang XF [19]	2015	China	BRC	92	qRT-PCR	Mean	43	3	40	35	8	49	15	34	19	30	OS	Yes	Rep	2.891 (1.483–6.215)	60 (Total)
He X [20]	2014	China	CC	48	qRT-PCR	Median	24	6	18	19	5	24	15	9	9	15	OS	No	SC	1.92 (0.81–4.55)	40 (Total)

CCA: cholangiocarcinoma, ESCC: esophageal squamouscell carcinoma, HCC: hepatocellular carcinoma, GC: gastric cancer, BRC: breast cancer, CC: colon cancer, qRT-PCR: quantitative real-time polymerase chain reaction, LNM: lymph node metastasis, TNM: tumor stage, LTS: low tumor stage(I, II), HTS: high tumor stage(III, IV), OS: overall survival, NA: not available, Rep: reported, SC: survival curve.
